# Cardiovascular and Hepatic Toxicity of Cocaine: Potential Beneficial Effects of Modulators of Oxidative Stress

**DOI:** 10.1155/2016/8408479

**Published:** 2015-12-28

**Authors:** Manuela Graziani, Letizia Antonilli, Anna Rita Togna, Maria Caterina Grassi, Aldo Badiani, Luciano Saso

**Affiliations:** ^1^Department of Physiology and Pharmacology “Vittorio Erspamer”, Sapienza University of Rome, Rome, Italy; ^2^Drug Addiction and Clinical Pharmacology Unit, University Hospital Umberto I, Sapienza University of Rome, Rome, Italy; ^3^Sussex Addiction Research and Intervention Centre (SARIC), School of Psychology, University of Sussex, Brighton BN1 9RH, UK

## Abstract

Oxidative stress (OS) is thought to play an important role in the pharmacological and toxic effects of various drugs of abuse. Herein we review the literature on the mechanisms responsible for the cardiovascular and hepatic toxicity of cocaine with special focus on OS-related mechanisms. We also review the preclinical and clinical literature concerning the putative therapeutic effects of OS modulators (such as N-acetylcysteine, superoxide dismutase mimetics, nitroxides and nitrones, NADPH oxidase inhibitors, xanthine oxidase inhibitors, and mitochondriotropic antioxidants) for the treatment of cocaine toxicity. We conclude that available OS modulators do not appear to have clinical efficacy.

## 1. Introduction

Oxidative stress (OS) can be defined as an unbalance between the production of reactive oxygen and nitrogen species (ROS and RNS) and the compensatory response of physiological antioxidant mechanisms. The main ROS are superoxide (O_2_^∙−^), hydrogen peroxide (H_2_O_2_), hydroxyl (^∙^OH), hydroperoxyl (HO_2_^∙^), peroxyl (RO_2_^∙^), alkoxyl (RO^∙^), singlet oxygen (^1^O_2_), hypochlorous acid (HOCl), and ozone (O_3_) while the main RNS are nitric oxide (^∙^NO), nitrogen dioxide (^∙^NO_2_), peroxynitrite (ONOO^−^), nitrous acid (HNO_2_), dinitrogen tetroxide (N_2_O_4_), dinitrogen trioxide (N_2_O_3_), and nitronium cation (NO^2+^). The most important sources of ROS and RNS are represented by enzymatic reactions localized in the mitochondria, the microsomes (cytochrome P450 enzymes), the cytosol such as xanthine oxidase (XO), and the membrane-associated protein complex with its cytosolic subunits NADPH oxidase (Nox). The production of ROS in the phagocytes depends on the activity of peroxidases such as myeloperoxidase and eosinophil peroxidase.

It has been suggested that OS plays an important role in the physiopathology of various apparatuses and organs including the cardiovascular system (ischemia and reperfusion injury, heart failure, atherosclerosis, hypertension, etc.) and the liver (acute and chronic damage) [1, 2]. There is also evidence of significant involvement of OS in the pharmacological and toxic effects of drugs of abuse and particularly of psychostimulants such as cocaine and methamphetamine [3].

The level of OS in the aforementioned conditions can be measured by a number of biomarkers, including H_2_O_2_, NO derivatives (nitrite, nitrate, and S-nitrosothiols), isoprostanes (deriving from the peroxidation of arachidonic acid), MDA and other thiobarbituric acid reactive substances (TBARS), 4-hydroxynonenal (4-HNE), acrolein, thiol/disulfide ratio, oxidation products of DNA (8-hydroxy-2-deoxyguanosine, 8-OH-G) and RNA (8-hydroxyguanosine, 8-OHD), and nitrotyrosine. It is of note that, in several studies, cocaine-induced OS was evaluated by the measurement of TBARS [4–9] which is considered inferior to other methods for lipid peroxidation like the evaluation of F2-isoprostanes [10].

In the present paper, we review the literature concerning the cardiovascular and hepatic toxicity of cocaine with special attention to the role of OS and the evidences about the possible modulators of OS which could have beneficial effects in cocaine users.

## 2. Cardiovascular Toxicity of Cocaine

The earliest case reports of cardiovascular toxicity attributed to cocaine date from the 1980s [11–13]. Cocaine abuse is associated with both acute and chronic cardiovascular toxicity [14–16], including myocardial ischemia [13, 17] and infarction [18], arrhythmias [19], and cardiomyopathy [20–22]. Recent epidemiological data indicate that cocaine is responsible for a sizeable proportion of emergency department visits and of sudden deaths [23, 24]. Data from 19 European countries indicated more than 500 cocaine-related deaths in 2012 [25]. Approximately 5% to 10% of emergency department visits in the United States have been attributed to cocaine-acute toxicity, chest pain being the most common symptom [15]. The upward trend in cocaine-related chest pain and myocardial infarction cases has induced the America Heart Association to draft diagnostic and therapeutic guidelines [26]. Data from the relative National Cardiovascular Data Registry was recently published [27]. Histopathological studies have shown that cocaine can precipitate myocardial ischemia in the presence of coronary artery occlusion [28] as well as of normal coronary arteries [29]. A recent review [23] of 49 cocaine-related deaths identified coronary atherosclerosis, ventricular hypertrophy, cardiomegaly, myocarditis, and contraction band necrosis in almost a third of cases.

The pathogenesis bases of cocaine-induced cardiovascular toxicity [14, 30, 31] have been studied in detail [32, 33]. Cardiovascular cocaine toxicity can be related to its pathophysiological effects on the sinoatrial node, myocardium, and vasculature, including the coronary district.

### 2.1. Pathogenetic Mechanisms of the Cardiac Toxicity of Cocaine

Cocaine can damage the heart through a variety of mechanisms that have been elucidated only in part. In the first place, cocaine has a direct cardiotoxic effect, due its ability to block voltage-dependent K+ and Na++ channels in the sinoatrial node and the myocardium, leading to reduced contractility and to prolongation of the QT interval and the QRS complex. It has been proposed that these two effects may produce acute myocardial ischemia and infarction also in absence of long-term cocaine abuse, of abnormalities in the coronary arteries, and of other risk factors [34, 35].

Cocaine can exert its toxic effect on the heart also indirectly, through the actions of catecholamines, and in particular of norepinephrine. Indeed, cocaine is known to block the reuptake of catecholamines by binding the transporters for dopamine (DAT) and norepinephrine (NET) [36]. Increased norepinephrine levels in the terminals of the sympathetic nervous system lead to activation of adrenergic receptors. Activation of *β*1 adrenergic receptors located on the cells of the sinoatrial node and of the myocardium results in increased heart rate and contractility. Activation of *α*1 receptors located on the smooth muscle cells of blood vessels leads to vasoconstriction and increased blood pressure. Increased heart rate, heart contractility, and blood pressure may lead to an acute imbalance in oxygen supply/demand [37, 38]. Oxygen deficiency can be further exacerbated by the reduction in blood supply to myocardium produced by *α*1-mediated constriction of coronary arteries [13]. In turn, oxygen deficiency may lead to myocardial infarction. Furthermore, the combination of the direct toxic effect of cocaine and those of norepinephrine may lead to complex arrhythmia [39].

In addition to producing oxygen imbalance, catecholamines may damage the myocardium via at least three additional pathogenetic mechanisms [30, 40]. First catecholamines can promote cation translocation from the vascular space to intracellular compartment [41] thus reducing serum K+ [42] and Mg 2+ [43]. Concurrent hypokalaemia and hypomagnesaemia may lead, given the pivotal role of these cations in activity of the Na+/K+-ATPase pump [44], to further cation dyshomeostasis, which in turn may contribute to apoptosis and necrosis of cardiomyocytes. Moreover, this mechanism [45], adding to the direct arrhythmogenic effects of cocaine described above, may facilitate the development of arrhythmias such as atrial fibrillation and ventricular tachycardia.

A second pathway responsible for cardiotoxicity is related, as first hypothesized by Fleckenstein and Coworkers in 1974 [46], to catecholamines-induced calcium overload in cytosol and mitochondria of cardiomyocytes [41]. Indeed, stimulation of *β*-adrenergic receptors leads to activation of protein kinase A (PKA) and increased Ca^2+^ levels in the cytosol. This leads to phosphorylation of Ca^2+^-protein substrates, including phospholamban, L-type calcium channel, ryanodine receptor, cardiac troponin I, and myosin-binding protein C [47, 48]. Increased cytosolic Ca^2+^ triggers the release of Ca^2+^ in the mitochondria. The mitochondrial role in physiological intracellular Ca^2+^ homeostasis as well as in necrosis and apoptosis signaling is well demonstrated [49]. Mitochondrial Ca^2+^ overload impairs respiration and ATP production and produces change in permeability of the mitochondrial membrane (eventually leading to its rupture), which represents critical events for the further structural degeneration of cardiomyocytes [50].

Moreover Ca^2+^ overload (as well as ROS mitochondrial production, see below) is responsible for the massive opening of mitochondria Permeability Transition Pores (mPTP) [51] resulting in further dysfunctional and structural degeneration of these organelles.

An indirect cardiotoxic effect of catecholamines may derive from action of their oxidation products, the aminochromes. Notably, excessive level of circulating catecholamines (and consequent saturation of the monoaminooxidase and catechol-o-methyl transferase systems) may cause the increased formation of adrenochrome (obtained by oxidation of adrenaline) [40, 52] of 5,6-dihydroxy-1-methylindole and of adrenochrome alkaline rearrangement product adrenolutin. In the heart the enzyme cytochrome c oxidase has been associated with adrenochrome formation [52].

Experimental studies investigating the relationship between aminochromes and cardiotoxicity [52, 53] demonstrated a direct toxic effect on cardiomyocytes: disturbances in cellular Ca^2+^ homeostasis and a perturbation of oxidative phosphorylation have been reported. Accordingly, increased levels of adrenolutin were observed in death-associated heart failure [54]. Moreover, aminochromes are known to induce redox cycling with consequent generation of ROS (see below).

More recently, OS and generation of ROS have been identified as one of the most important mechanisms of cocaine-induced cardiomyocyte toxicity [6, 30, 31, 55, 56]. Increased expression iNOS and decreased levels of myocardial SOD and catalase were found in the cardiomyocytes of patients with dilated cardiomyopathy related to chronic cocaine abuse [20]. Indeed, ROS formation has been thought to be related to cocaine-induced catecholamine release [56]: *α*_1_ and *β* receptors stimulation, as well as enzymatic and nonenzymatic degradation of catecholamines, lead to intracellular ROS formation.

#### 2.1.1. *α*_1_-Adrenoceptors Stimulation and Production of ROS

In the plasma membranes of cardiac cells, *α*_1_-adrenoceptors stimulation increases the activation of nicotinamide adenine dinucleotide phosphate (NADPH) oxidases [57], an electron donor which in turn produces the superoxide anion radical O_2_^∙−^ formation [56, 58]. The role of NADPH-driven superoxide production in cocaine induced cardiac dysfunction is well recognized [59]. Indeed, the family of Nox enzymes is considered the major sources of ROS in the cardiovascular system [58, 60]: the proteins of the Nox family produce O_2_^∙−^ by transferring an electron from NADPH (or NADH) to O_2_. It is believed that Nox1, Nox2, and Nox4 are expressed significantly in the vascular system [61, 62] while experimental data in cardiomyocytes had demonstrated that the Nox subunit Nox1, but not Nox2 or Nox4, is implicated in norepinephrine-induced Nox-generated ROS [63]. Inhibition of Nox activity by apocynin prevented the increase in ROS production and cardiac dysfunction induced by chronic administration of cocaine* in vivo* in rats [64]. Moreover, Nox activity is associated with other ROS-inducing enzymatic sources such as xanthine oxidoreductase (XOR): a fundamental role of XO was confirmed in an* in vivo* experimental model of cocaine-induced diastolic dysfunction [65], in which treatment with XO-inhibitor allopurinol prevented the cocaine-increase in mitochondrial ROS levels.

#### 2.1.2. *β*-Adrenergic Receptors Stimulation and Production of ROS

As discussed above, *β*-adrenergic receptors (*β*-AR) stimulation activates a GTP-binding protein S, which stimulates adenylyl cyclase (AC) to produce cAMP, which in turn activates PKA. It has been demonstrated that *β*-AR stimulation may contribute to mitochondrial ROS production in cardiomyocytes: indeed the *β*-adrenergic agonist isoproterenol (ISO) increased, in a concentration- and cAMP-protein kinase A-dependent manner, mitochondrial O_2_^∙−^ production in freshly isolated mouse cardiomyocytes and induced a twofold increase of MDA protein adducts relative to controls in perfused hearts [66]. Accordingly,* in vivo* administration of mitochondria-targeted antioxidant MitoQ had shown to prevent left ventricular (LV) diastolic dysfunction (characterized by an increase in the index of LV relaxation, in LV end-diastolic pressure-volume relation, and in LV end-diastolic pressure) induced in rats treated with cocaine for 7 days [67]. Beta-AR stimulation induced both cAMP [68] and PKA increase both in bovine [69] and in rat [68, 70] cardiomyocytes mitochondria.

Experimental data in mouse cardiomyocytes [66] have demonstrated that *β*-AR exposure to agonist ISO leads to an increase in ROS, suggesting ROS production as a direct consequence of activation of the cAMP-PKA signaling pathway in mitochondria. Importantly, the increase in mitochondrial ROS production appeared to be Ca^2+^-independent, since a selective increase of the amplitude of Ca^2+^ transient did not increase ROS production [66].

*β*-AR stimulation is also implicated in the impairment of antioxidant system. Indeed, significant reduction in CuZn-SOD enzyme activity has been found in hearts from ISO-treated rats as well as in ISO-stimulated isolated cardiomyocytes [71]. Moreover, a reduction in manganese-SOD (Mn-SOD) expression induced by chronic ISO was more recently found in type 5 AC (1 of 2 major AC isoforms in heart) transgenic mice [72].

#### 2.1.3. OS from Catecholamines Metabolites (Aminochromanes)

As mentioned above, when the enzymatic catabolism of catecholamines is not sufficient, they can undergo chemical oxidation causing additional OS [40]. The implication of aminochromanes in the pathogenesis of cardiac diseases [53, 73] is also well recognized.

Formation of superoxide anion O_2_^∙−^ (due to an oxidation pathway that involves the formation of highly reactive intermediaries* o*-semiquinones and* o*-quinones) [74] has been observed in both* in vivo* [75] and* in vitro* models [74, 76]. The superoxide anion O_2_^∙−^ can cause the oxidation of epinephrine [52, 77, 78] and of metal ions copper [79] and iron [77], enhancing the oxidation of catecholamines. Thus, iron chelation can protect against the cardiotoxicity induced by the metabolites of catecholamines: an* in vitro* study in rat ventricular cardiomyoblast assessing the potential cardioprotective effects of some chelating agents had suggested further investigation* in vivo* animal models [80].

Catecholamines metabolites can also reduce antioxidant defences by decreasing the levels of reduced glutathione (GSH) and increasing the oxidized glutathione (GSSG) content as observed in adrenaline-treated isolated rat cardiomyocytes [74, 79].

### 2.2. Pathogenetic Mechanisms of Cocaine Vascular Toxicity

#### 2.2.1. Cocaine Effect on Vascular Smooth Muscle Cells

Cocaine sympathomimetic activity and consequent agonist action at *α*_1_ adrenergic receptors (*α*_1_-AR) in vascular smooth muscle cells cause contraction in the vascular system. The activation of these receptors leads in fact to the formation of inositol triphosphate and diacylglycerol (via phospholipase C), which in turn cause an increase in Ca^2+^ entry and in release from Ca^2+^ stores in smooth muscle cells [81]. Despite the different contribution of each *α*_1_-AR subtype [82, 83] in the regulation of contraction in different vascular districts, the net result of *α*_1_-AR stimulation is represented by an acute increase in blood pressure.

#### 2.2.2. Cocaine Effect on Endothelial Cells

A further contribution in the pathogenesis of cocaine-induced vasoconstriction is due to its acute and chronic effect on endothelial cells function [84]. An impairment in endothelium-dependent vasorelaxation, assessed as a decrease in forearm blood flow in response to intraarterial acetylcholine and nitroprusside, was found in long-term users of cocaine [85]. Accordingly experimental studies [86, 87] demonstrated a cocaine-induced endothelial dysfunction with a decrease in NO release and in the constitutive enzyme NO-synthase (eNOS) content, as well as an increase in endothelin-1 (ET-1) production and ET-1 receptor type-A (ET_A_R) protein expression [84]. Increased number of circulating endothelial cells (CECs) indicating endothelial dysfunction was recently demonstrated in cocaine abusers [88]. Furthermore, enhancement of cocaine-induced vasoconstriction was observed after N(G)-nitro-L-arginine methyl ester-induced inhibition of NO synthesis* in vitro* [89].

Concomitant decrease in NO-induced vasodilatation and ET-1-induced vasoconstriction may enhance *α*_1_-AR-mediated vasoconstriction. It has been suggested that inhibition of eNOS expression may partially derive from high levels of ET-1 through a PKC-mediated pathway in endothelial cells [90] and/or through ET-1 receptor type-A (ET_A_R) stimulation, which in turn can increase ROS production [91, 92].

Chronic cocaine exposure and consequent endothelial dysfunction may precipitate early atherosclerosis [93] and the persistence of endothelial cell damage beyond its acute effect on the blood vessels can further increase cardiovascular risk in cocaine abusers [88, 94]. Since that ET-1 increase was significantly associated with atherosclerotic lesion [95], it may be argued that it plays a fundamental role in the cocaine-induced vasoconstriction at sites of significant stenosis [96].

Although discrepant results were also reported [97], probably due to methodological differences, there is some evidence indicating that cocaine may exert an inhibitory effect on the production of prostacyclin (PGI_2_) by endothelial cells [98, 99]. Since the release of both vasodilatatory PGI_2_ and NO may result from stimulation of ET-1 receptor type-B_1_ (ETB_1_) receptors on endothelial cells, it is possible that the inhibitory effect of cocaine on NO release also extends to PGI_2_ release [100].

Another important factor that can contribute to cocaine prothrombotic action is its direct action on endothelial cells secretion of von Willebrand factor (VWF). Indeed, a recent* in vitro* study [101] demonstrated that cocaine and its metabolites induced VWF secretion in a concentration-dependent manner from three endothelial cell types (human umbilical vein, brain microvasculature coronary artery endothelial cells).

#### 2.2.3. Cocaine Effect on Platelet Function

Whereas both experimental and clinical data agree on the fact that cocaine can affect endothelial function, leading to vasoconstriction, there is conflicting evidence about the direct effect of cocaine on platelets. Increased platelet activation was observed* in vitro* in rabbit platelet-rich plasma (PRP), preincubated with cocaine hydrochloride [102] as well as* in vivo* in dogs treated with intravenous cocaine [103]. The findings obtained with human platelets* in vitro* are less consistent. At concentrations comparable to the systemic concentrations produced by lethal doses, cocaine decreased platelet aggregation induced by agonists in PRP obtained from healthy human subjects [104]. In contrast, two* in vitro* studies conducted with cocaine concentrations comparable to the systemic concentrations associated with the “high” showed increased platelet activation in whole blood preparations [105, 106], whereas other studies found no effect of cocaine [107]. Finally, increased platelet expression of surface P-selectin was found in blood samples from chronic cocaine users [107]. It is possible that these discrepancies were due to differences in substrate (animal versus human platelets), in model (*in vitro* versus* in vivo*), in methodology (whole blood versus PRP), and in the concentrations of cocaine used.

*In vivo* studies in healthy subjects [108] and in chronic cocaine user [109], although not univocally [110], gave evidence of activation of platelets, assessed by increase of P-selectin expression [109] and of soluble CD40L, a transmembrane molecule mainly expressed by activated platelets [111]. Due to physiological interaction of platelets with vascular endothelium and circulating blood cells such leukocytes, it may be argued that an indirect, rather than a direct, mechanism of action is involved in the cocaine platelets activation. Indeed, the above mentioned cocaine-induced vasospasm, consequent increase in shear stress, and endothelial dysfunction may induce a platelet activation, as demonstrated by release of constituents of their *α*-granules [106] and of thromboxane A2 [99]. A further contribution may derive from VWF interaction with the platelet receptor GPIb and their subsequent activation [108].

#### 2.2.4. OS and Cocaine-Induced Endothelial Toxicity

Besides cardiomyocytes, also endothelial cells (ECs) may release ROS, mainly due to its presence of enzymes such as XO, NOS, mitochondrial MAO, and NAPDH oxidase. Some data in literature indicate a direct or an indirect cocaine action on endothelial cells, both on enzymes expression and on mitochondrial function.

#### 2.2.5. XO and Production of ROS

In bovine aortic endothelial cells, it has been observed that shear stress induced an enhancement of xanthine-dependent O_2_^∙−^ production [112], associated with a decrease in xanthine dehydrogenase (XDH) protein. Furthermore, inhibition of the Nox decreased XO levels and prevented the increase of O_2_^∙−^, highlighting the central role of Nox in modulating endothelial production of ROS. It may be suggested that vasoconstriction effect of cocaine and consequent blood shear stress may trigger endothelial ROS production via Nox/XO enzyme.

In agreement with this hypothesis, cocaine-induced cardiac dysfunction (alteration of cardiac output and stroke volume) was found to be associated with increased Nox and XOR activity* in vivo* study in rats [64]. Furthermore, apocynin or allopurinol treatment inhibited the cocaine-induced cardiac alteration and the myocardial production of O_2_^∙−^ confirming the role that Nox-derived ROS play in modulating ROS production by XO [64].

#### 2.2.6. Nitric Oxide Synthase and Production of ROS

As mentioned above, cocaine-induced decrease in endothelial NO release and in the constitutive enzyme eNOS content has been observed [84]. A contribution to this cocaine endothelial toxic effect may derive from its increasing action in Nox, which in turn (besides the increase in O_2_^∙−^) may induce oxidation of tetrahydrobiopterin, a cofactor of NO synthase: as a consequence eNOS uncoupling leads to the observed reduction in NO synthesis and to an enhancement in O_2_^∙−^ [113].

#### 2.2.7. ROS in Endothelial Mitochondria

Besides the well-recognized energy-producing activity, notably mitochondria are the major source of cellular reactive oxygen [114] both in cardiomyocytes and in EC [40, 115]. An important role of endothelial mitochondria in pathogenesis of endothelial dysfunction is due to their role in signaling cellular responses, among which the production of ROS [116].

#### 2.2.8. Mitochondrial Monoaminooxidase and Production of ROS

Among the sources of mitochondrial ROS, the monoamine oxidase (MAO) family, located to the outer mitochondrial membrane, causing the oxidative deamination of catecholamines, results in hydrogen peroxide (H_2_O_2_) formation [117]. Accordingly, experimental data in literature indicate an increase in H_2_O_2_ cardiac production after the sympathomimetic drug amphetamine administration [118]. In this regard a recent* in vitro* study has demonstrated in human pulmonary EC [119] exposed to cocaine a significant increase in H_2_O_2_ production; due to the modulating action of H_2_O_2_ on endothelium functions such as endothelium-dependent vasorelaxation, apoptosis, and remodeling [120] it may be argued that cocaine-induced increase in catecholamines and in the consequent MAO-mediated H_2_O_2_ production in endothelial mitochondria could further enhance endothelial dysfunction, contributing to cocaine toxic effects on vascular district.

An important contribution to OS may derive from the process, namely, ROS-induced ROS release (RIRR) [120]: this process makes a significant amplification to ROS production [121]. Briefly, OS in the mitochondria may trigger the opening of mitochondrial transition pore that leads to a further increase in ROS generation. RIRR phenomena have been recognized in both physiological (promoting an elevation in the cell tolerance to OS, until the destruction of impaired-function mitochondria) [122] and pathological conditions such as cardiac ischemia-reperfusion [123] associated with acute myocardial infarction.

#### 2.2.9. ROS Production by Mitochondrial Nox4

Another possible source of ROS in endothelial mitochondria is Nox4 [124], generating a higher hydrogen peroxide to superoxide ratio than Nox1 and Nox2. Nox4 involvement in endothelial cells process and in responses to hypoxia and OS is well recognized [125]; moreover it has been demonstrated that the expression or activity of Nox4 is increased in response to the proinflammatory mediators TNF-*α* [126]. While a cocaine-induced increase in Nox has been demonstrated in cardiac tissue [64], to date no data in literature are present on the effects of cocaine on Nox at endothelial level. An indirect effect of cocaine activation of Nox may derive from the cocaine induction of the TNF-*α* expression, observed in bovine aortic endothelial cells [127]. Moreover a Nox increase and the consequent endothelial superoxide production were also found in bovine aortic endothelial cells exposed to oscillatory shear stress [112].

## 3. Cocaine Hepatotoxicity

Cocaine abuse is known to induce acute [128–132] and chronic [130, 131, 133] liver toxicity. Clinical manifestations of cocaine-induced hepatic damage range from elevation of liver enzyme levels in chronic users [131, 133, 134] to acute liver failure associated with hepatitis [128], to fulminant liver failure associated with acute rhabdomyolysis [132] or thrombotic microangiopathy [128]. Histopathological examination has shown midzonal [132] and periportal [130] necrosis, as well as steatosis in the surviving hepatocytes [131]. The involvement of OS in cocaine liver toxicity has been reviewed recently [135, 136].

*In vivo* animal studies suggest the involvement of cocaine metabolites in the genesis of hepatotoxicity. Experiments conducted in rats [137] and mice [138, 139] have shown that cocaine-induced hepatotoxicity is at least partly dependent on cocaine N-demethylated metabolites norcocaine (NCOC), and N-hydroxynorcocaine (N-OH-NCOC) and norcocaine nitroxide (NCOC-NO_∙_) [139, 140]. Inhibition of cytochrome P-450 (CYP450) mediated activation of cocaine metabolism produced a significant inhibition of hepatotoxicity in mice* in vivo* [138] and* ex vivo* in liver microsomes [141], while* in vivo* induction of CYP450 activity enhanced the cocaine hepatotoxicity [142]. Studies in mice demonstrated that cocaine reduces GSH levels in the liver [143, 144] and that depletion of intracellular GSH concentrations exacerbated cocaine hepatotoxicity [144]. Finally, it has been shown that pretreatment with the GSH precursor NAC exerted a protective effect against cocaine hepatotoxicity in mice [145], while pretreatment with endotoxin lipopolysaccharide (LPS) led in Kuppfer cells to the formation of NO which exacerbated cocaine toxicity [146].

Given that the synthesis of both NCOC-NO_∙_ and GSH requires the presence of the cofactor NADPH, it has been hypothesized that a decrease in hepatocytes NADPH content and consequent impairment of antioxidant system may contribute to enhance OS [147].

The ability of cocaine to deplete intracellular GSH may also depend on the effects of its N-oxidative metabolites on the mitochondrial function of the hepatocytes.* In vivo* studies in rats [148, 149] have shown that these metabolites can decrease GSH levels and membrane potential and increase ROS production in the mitochondria [9]. Mitochondrial generation of ROS has been shown also in isolated mouse liver mitochondria treated with NCOC, N-OH-NCOC, and NCOC-NO_∙_ but not with cocaine [150], confirming the fundamental role of the N-oxidative metabolites of cocaine in OS-mediated hepatotoxicity.

*Ex vivo* and* in vitro* studies have confirmed the* in vivo* data. Cocaine-induced GSH depletion and GSSG production were observed in cultures of mouse and, to a lesser extent, in rat cocaine-treated hepatocytes [151, 152] with mouse being more sensitive to cocaine hepatotoxicity, as observed in cultured liver slices from different species [153]. In primary cultures of rat hepatocytes treated with the CYP-450 inducer phenobarbital, it has been observed that cocaine-induced alteration in the thiol redox equilibrium may be crucial for the development of hepatocyte toxicity and consequent LDH release [154]. Moreover cocaine-treated hepatocytes had been associated with decrease in catalase and Mn-SOD [155]. Further confirmation of implication of OS is derived from rat hepatocyte model of cocaine cytotoxicity in which a partial prevention of cytotoxicity by NAC [156] and by deferoxamine (a ferric iron chelator) was observed [152, 156].

In summary, human and experimental data provide evidence of direct toxic effects of cocaine on the hepatocytes. This hepatotoxicity appears to be dependent mainly on N-oxidative metabolites of cocaine. Impairment of the antioxidant system as depletion of intracellular and mitochondrial GSH also contributes to cocaine hepatotoxicity.

## 4. Potential Therapeutic Effects of OS Modulators in Cocaine Abuse

Antioxidants or modulators of OS as they are often referred to due to their possible prooxidant activity can be classified in low (LMW) and high (HMW) molecular weight and further subdivided into endogenous or exogenous. The exogenous ones can be natural or synthetic [2]. Several evidences indicate that some of these compounds could have beneficial effects on cocaine-induced toxic effects.

### 4.1. NAC

NAC has been used for many years in the treatment of acute paracetamol intoxication, as a mucolytic for chronic obstructive pulmonary disease and as a protectant in contrast-induced nephropathy. NAC can scavenge directly ROS and particularly the hydroxyl radical ^∙^OH and hypochlorous acid but due to its high first pass metabolism and low bioavailability acts mainly as a precursor of GSH in many organs, including the liver [145] and the brain [157]. As mentioned before, mice pretreatment with NAC had a protective effect against cocaine-mediated hepatotoxicity [146]. Importantly, NAC treatments appear to be safe and tolerable both in preclinical and in clinical studies [158, 159]. GSH is a very important endogenous antioxidant in the brain and it has been known for a long time that its level decreases in neuropsychiatric disorders such as depression, schizophrenia, and bipolar disorder [160] and is currently considered a promising drug for these pathological conditions [158, 160]. A recent double-blind placebo-controlled trial failed to demonstrate that NAC reduces cocaine use in cocaine-dependent subjects but showed some evidence that it can prevent relapse in individuals who had already achieved abstinence from cocaine [161]. Thus NAC may be useful as a relapse prevention agent in abstinent cocaine-dependent subjects [161].

The mechanism of action of NAC in the aforementioned conditions is complex and not completely elucidated. The main activities contributing to its efficacy seem to be related to (i) its capability of providing cysteine which is the limiting amino acid in GSH production; (ii) the exchange of extracellular cysteine provided by NAC with intracellular glutamate by the cysteine/glutamate transporter causing the activation of presynaptic mGluR2/3 receptors which inhibit glutamatergic neurotransmission and excitotoxicity [162, 163]; (iii) direct antioxidant activity related to its capability of scavenging radicals such as hydroxyl and peroxynitrite [164]; (iv) induction of the expression of antioxidant enzymes through the nuclear E2-related factor (Nrf2)/ARE system [165]. It is of note that the Nrf2 pathway represents a promising therapeutic approach to restore the redox balance in the CNS and in other organs and that only a few Nrf2-activating compounds have been tested in a clinical setting until now [166].

### 4.2. SOD Mimetics

SOD is a very important antioxidant enzyme in mammals existing in three different physiological forms: MnSOD in the mitochondrial matrix, CuZnSOD in the cytoplasm, and CuZnSOD in extracellular fluids [167]. Given the central role of the superoxide radical in oxidative pathological phenomena, different drugs with SOD activity were developed [168].

An infusible form of human manganese SOD (rMn-SOD) as new therapeutic option capable of crossing cell membranes was recently proposed for dilated cardiomyopathy caused by cocaine [20].

### 4.3. Nitroxides and Nitrones

Most antioxidants acting as spin traps have a nitroxide or nitrone nucleus.

Nitroxides are nonmetal catalytic antioxidants. One of the most common is Tempol (4-hydroxy-2,2,6,6-tetramethylpiperidine-N-oxyl) with SOD and catalase enzymatic activities which can be administered orally and can cross biological membranes and react with ROS intracellularly and within mitochondria [169].

Nitrones are potent antioxidants capable of forming stable nitroxyl radicals when reacting with oxygen radicals. Several nitrones, including *α*-phenyl-tert-butylnitrone (PBN), have been shown to have neuroprotective properties in experimental animal models of stroke, Parkinson's and Alzheimer's disease [170], as well as in preclinical models of CNS injury [171]. Treatment with Tempol can attenuate OS in the prefrontal cortex and in the nucleus accumbens [172] and inhibited cocaine self-administration* in vivo* in rats, suggesting that reinforcing effects of cocaine are mediated, at least in part, by ROS [173].

To the best of our knowledge, no data are present in literature on efficacy of nitroxides and nitrones on cocaine-induced cardiac or hepatic injury.

### 4.4. Nox Inhibitors

As mentioned above, Nox are among the best characterized sources of superoxide and the predominant ROS producing enzymes in the vascular smooth muscle cells, in the myocardium, and in blood vessels [174]. Hypertension, atherosclerosis, hyperlipidemia, and diabetes are associated with endothelial dysfunction probably mediated by Nox driven production of ROS [175]. Compared to radical scavengers, Nox inhibitors could be more effective because they are able to block the formation of ROS at the source [176].

Several Nox inhibitors are currently available, including apocynin, which is an orally active natural compound obtained from the roots of* Picrorhiza kurroa* capable of inhibiting Nox activation probably by preventing the assembly of Nox2 with p47phox [177, 178].

Importantly it was reported that apocynin can prevent cocaine-induced myocardial damage in rats [64] providing a good evidence for the therapeutic potential of Nox inhibitors in the treatment of cocaine cardiotoxicity [179].

### 4.5. XO Inhibitors

Allopurinol is a well-known inhibitor of XO used for decades for the treatment of gout [180]. It can prevent OS by inhibiting XO and also by scavenging directly hydroxyl free radicals.

Clinical studies showed that allopurinol has beneficial effects in chronic kidney disease patients [181]. XO inhibition by allopurinol (or the active metabolite oxypurinol) could be beneficial in hyperuricemic patients with congestive heart failure [182]. Unfortunately, allopurinol is not devoid of side effects such as severe skin reactions and renal impairment [182].

Allopurinol and 1,3-dimethylthiourea, a scavenger of hydroxyl radical, produced a potent inhibition of hepatotoxicity induced by cocaine in adult male mice [183].

Apocynin or allopurinol prevented cocaine-induced cardiac alteration by restoration of cardiac output, stroke volume, and fractional shortening, associated with a reduction of the myocardial production of superoxide anions and an enhancement of catalase activity [55] suggesting that NADPH and XO can act synergically to form myocardial ROS and that their inhibition could prevent the onset and progression of cocaine-induced left ventricular dysfunction [55].

### 4.6. Mitochondriotropic Antioxidants

Cationic triphenylphosphonium (TPP) derivatives such as MitoQ have high affinity for the inner mitochondrial membrane that is negatively charged. They can cross the blood-brain barrier even if the uptake in the brain appears less than for other organs [184]; TPP compounds can be administered orally and did not raise safety concerns so far [184]. MitoQ was protective in a large number of cell models of mitochondrial OS and has been shown to be well tolerated, orally active, and safe in humans [185].

Cocaine can be toxic for mitochondria, causing formation of ROS, impaired electron transfer, suppression of ATP production, membrane permeabilization with release of cytochrome C, and subsequent cell death: MitoQ can prevent these abnormalities protecting against cardiac dysfunction [67].

Preparation of small cell-permeable peptides (SS-peptides) which accumulate in the mitochondria and bind to the inner membrane thanks to the presence of basic amino acids positively charged at physiological pH had shown to protect mitochondria against oxidative damage [185, 186]. The use of these peptides to deliver antioxidant molecules into mitochondria and provide protection against cocaine induced OS has been proposed [187].

### 4.7. Deferoxamine

NAC and deferoxamine (DFO, an iron chelating agent that prevents ROS generation by inhibiting the Fenton reaction) protected rat hepatocytes in culture against the OS induced by cocaine, confirming the involvement of oxygen radicals in cocaine-induced necrosis/apoptosis [188]: hepatocytes were preincubated for 24 h either in the presence or in the absence of NAC or DFO and exposed for another 24 h to increasing concentrations of cocaine together with NAC or DFO. Pretreatment with deferoxamine complex with iron ion (III), aminoguanidine, and N-methyl-d-glucamine dithiocarbamate complex with iron ion (II) produced a marked inhibition of the hepatotoxicity induced by a single administration of cocaine, as indicated by histopathological examination, alanine aminotransferase activity, and nitrite/nitrate levels [183].

### 4.8. Selenium

In rats, pretreatment for 4 weeks with selenium reversed both the OS and the contractile dysfunction induced by repeated (7 days) cocaine administration in rats [189]: cardiac function was evaluated by cardiac index and left ventricular fractional shortening measured by echocardiography.

### 4.9. Minocycline

Minocycline, a second generation tetracycline, has been found in rats to prevent cardiac myocyte death (associated with an increase in OS evidenced by low GSH/GSSG ratio and increased levels of 4-hydroxy-2-nonenal) induced by prenatal cocaine exposure [190].

### 4.10. Auranofin

Pretreatment of mice with auranofin (a well-known disease-modifying gold compound used for rheumatoid arthritis recently proposed also for other therapeutic applications) [191] showed an interesting protective effect against hepatic injury caused by cocaine by inducing overexpression of heme oxygenase-1 (HO-1), an important OS marker responsible for heme degradation [192]. Unfortunately, auranofin is not devoid of toxic effects [193] but this result indicates that it could be worthwhile to search for less toxic inhibitors of HO-1 to reduce hepatic injury induced by cocaine.

### 4.11. Amiodarone

Amiodarone is a class III antiarrhythmic drug used in heart failure and postischemic heart capable of protecting cardiac myocytes against OS [194]. However, amiodarone pretreatment did not affect the seizure incidence or mortality in mice treated with high doses of cocaine [195]. The efficacy of amiodarone in the treatment of cocaine-induced dysrhythmias is still undefined [196] and further studies should be performed on this and other antiarrhythmic drugs.

### 4.12. Trolox

Trolox, a hydrophilic analog of vitamin E with well-known antioxidant properties [197], was found to reduce the production of ROS induced by exposure to cocaine and the neurotoxic effect of the HIV-1 transactivating protein Tat [198].

## 5. Conclusion

In the present paper, several studies were reported demonstrating the important role of OS in the activity and toxicity of cocaine with special attention to its cardiovascular and hepatic effects. Several evidences were reported showing the potential beneficial effects of antioxidants or modulators of OS (as they are referred to considering their possible prooxidant effects in certain conditions) even if it should be kept in mind that, like in the case of NAC, their mechanism of action, which is often complex and not always fully elucidated, is not only related to their antioxidant activity. Unfortunately, for most of these modulators of OS the preclinical and clinical studies are limited and do not allow drawing definitive conclusions on their efficacy in relation to cocaine toxicity. Even in the case of NAC limited clinical trials were performed in cocaine users and the biomarkers of OS were not considered with sufficient attention.

Other studies should then be performed also considering the difficulty to demonstrate the clinical efficacy of modulators of OS. In fact, several clinical trials on antioxidants in pathological events such as neurodegenerative, cardiovascular, or pulmonary diseases, conditions associated with ischemia and reperfusion injury, chronic kidney disease, or psychiatric disorders failed in the last few years for different possible reasons including [1, 2]:

1. In physiological conditions, OS is controlled by a complex mixture of endogenous and exogenous antioxidants (*lipophylic* or hydrophilic, enzymatic or nonenzymatic) and when used therapeutically one single antioxidant may not be sufficient. The challenge is then to design trials with the right mixtures of substances at the right doses.
2. Certain conditions probably require the delivery of higher amounts of antioxidants to specific organs or tissues or organelles such as the mitochondria.
3. The dose and/or duration and/or regimen and/or timing of the treatment should be appropriate.
4. Some antioxidants may have prophylactic activity but not be very effective after the onset of the condition. If the stage of the disease is too advanced, patients will not probably receive benefit from the therapy.
5. Most clinical trials were performed without a proper selection of the patients based on validated biomarkers of oxidative stress. Furthermore, whole body OS markers may not be appropriate for specific diseases in which OS involves specific tissues or organs or organelles.
6. Some antioxidants can act as prooxidants in certain conditions or have adverse side effects.

In conclusion the effect of modulators of OS on the activity and toxicity of cocaine should be further studied in order to develop new drugs useful for the treatment of OS-mediated cocaine toxic effects.

## Abbreviations

CNS: Central nervous system
GSH: Glutathione
HO: Heme oxygenase
MDA: Malondialdehyde
NAC: N-Acetylcysteine
Nox: NADPH oxidase
NO: Nitric oxide
OS: Oxidative stress
RNS: Reactive nitrogen species
ROS: Reactive oxygen species
SOD: Superoxide dismutase
XO: Xanthine oxidase.
